# Linac based SBRT for prostate cancer in 5 fractions with VMAT and flattening filter free beams: preliminary report of a phase II study

**DOI:** 10.1186/1748-717X-8-171

**Published:** 2013-07-08

**Authors:** Filippo Alongi, Luca Cozzi, Stefano Arcangeli, Cristina Iftode, Tiziana Comito, Elisa Villa, Francesca Lobefalo, Pierina Navarria, Giacomo Reggiori, Pietro Mancosu, Elena Clerici, Antonella Fogliata, Stefano Tomatis, Gianluigi Taverna, Pierpaolo Graziotti, Marta Scorsetti

**Affiliations:** 1 Istituto Clinico Humanitas Cancer Center, Radiotherapy and Radiosurgery, Rozzano, Milan, Italy; 2 Oncology Institute of Southern Switzerland, Medical Physics, Bellinzona, Switzerland; 3 Istituto Clinico Humanitas Cancer Center, Urology, Rozzano, Milan, Italy; 4 Humanitas Cancer Center, Istituto Clinico Humanitas, Via Manzoni 56, 20089, Rozzano, Milano, Italy

**Keywords:** Prostate, RapidArc, Stereotactic body radiation therapy

## Abstract

**Background:**

To evaluate the feasibility and early side effects of a short course hypo-fractionated SBRT programme with Volumetric Modulated Arc Therapy (VMAT) and Flattening Filter Free (FFF) beams.

**Methods:**

A prospective phase I-II study, started on February 2012. Inclusion criteria were: age ≤ 80 years, WHO-PS ≤ 2, PSA ≤ 20 ng/ml, histologically proven prostate adenocarcinoma, T1-T2 stage, no distant metastases, no previous surgery other than TURP, no malignant tumours in the previous 5 years, IPSS 0–7. The schedule was 35 Gy in 5 alternative days. SBRT was delivered with RapidArc VMAT, with 10MV FFF photons. Toxicity assessment was performed according to CTCAE v4.0 scale. EPIC questionnaires assessed Quality-of-Life. Neo-adjuvant/concomitant hormonal-therapy was prescribed according to risk classification. SpaceOAR™ gel was optionally implanted to increase the separation space between the prostate and the rectal wall.

**Results:**

Median follow-up was 11 months (range: 5–16); 40 patients were recruited in the protocol and treated. According to NCCN criteria, 26/40 patients were low-risk and 14/40 were intermediate risk. Median age was 70 years (56–80), median initial PSA was 6.25 ng/ml (0.50-13.43 ng/ml). Median Gleason score was 6 (6–7). All patients completed the treatment as programmed (median 11.8 days (9–22). Acute Toxicities were as follow: Rectum G0: 30/40 cases (75%); G1: 6/40 (15%); G2: 4/40 (10%). Genito-urinary: G0: 16/40 (40%); G1: 8/40 (20%); G2: 16/34 (40%). In two G2 urinary retention cases, intermittent catheter was needed. No acute G3 or greater toxicity was found. Median treatment time was 126 sec (120–136). SpaceOAR™ was implanted in 8 patients. PSA reduction from the pre-treatment value of the marker was documented in all patients.

**Conclusions:**

Early findings suggest that SBRT with RapidArc and FFF beams for prostate cancer in 5 fractions is feasible and tolerated in acute setting. Longer follow-up is needed for assessment of late toxicity and outcome.

## Background

In organ-confined prostate cancer, radical prostatectomy is the most common therapeutic procedure for disease eradication. Various radiation therapy (RT) techniques have been considered an effective non-invasive treatment option, especially for elderly patients and/or those unfit for surgery. Historically, brachytherapy has played a significant role in the radical treatment of prostate cancer, particularly in the subgroup of low-risk patients with a small prostatic gland and good urinary flow. Brachytherapy irradiates the prostatic gland through an intense intra-prostatic dose while minimizing irradiation of organs at risk, and is especially good at sparing the anterior rectal wall, and the urethra and bladder neck. Recent innovations in external beam radiotherapy (EBRT) which combine image-guided radiotherapy (IGRT) and intensity-modulated radiotherapy (IMRT), allow for delivery of an increased dose to the target while limiting toxicity to normal tissues. These innovations provide benefits that are similar to brachytherapy but with the additional advantage of being non-invasive. Nevertheless, in the context of definitive local treatment of prostate cancer, the total duration of a conventional RT course remains a critical issue for patients because the standard EBRT course usually lasts from 7 to 9 weeks.

Several studies of RT in prostate cancer suggest that prostate tumours may have a low alpha/beta ratio (estimated to approximately 1.5-2 in prostate versus 3 in the rectum) suggesting that the slow proliferating prostate cancer cells have high sensitivity to dose per fraction.

If low alpha/beta ratio is really low, inferior or equal to surrounding normal tissue, the linear/quadratic model suggests that Stereotactic Body Radiation Therapy (SBRT) delivered in few fractions of focused high doses should improve the therapeutic ratio in radiotherapy for prostate cancer. Compared to the use of stereotactic radio-surgery for other tumour sites, adoption of SBRT in the management of genitourinary malignancies has been slow. Nevertheless, emerging data are showing the safety and efficacy of this treatment modality in prostate cancer [1].

Stereotactic body radiation therapy (SBRT) with FFF (Free Flattened Filter) beam is a novel treatment modality that delivers a very high dose of precise radiation to the tumour target in a single or small number of fractions. This modality is safe and effective in both early stage primary cancer and oligo-metastases [2].

Here, we presented a phase II study designed to evaluate the safety of SBRT delivered in 5 fractions on prostate with or without seminal vesicles, by means Rapid Arc technique with Flattening Filter Free (FFF) beams. Primary end point of the current analysis was to evaluate the technical feasibility in terms of dosimetric point of view and the incidence of acute and early late complications in the first 40 low-intermediate risk prostate cancer patients.

## Methods

This is a prospective phase I-II pilot feasibility study, approved in 2012 by internal ethical committee.

### Patient population

From February 2012, the recruitment for low-intermediate risk prostate cancer patients was opened. The main objective was to study early and late side effects of hypo-fractionated accelerated RT for prostate cancer with FFF beam. The schedule chosen was 5×7 Gy = 35 Gy delivered in 5 alternative days, corresponding to an NTD2 between 70 and 85 Gy for an α/β estimate between 3 and 1.5 Gy. This fractionation avoided an accelerated schedule, possibly too intense for the acute mucosal tolerance (the estimated BED for acute mucosal reactions for the schedule of 7 Gy in 10 elapsed days is 56.7 Gy 10, below 63 Gy 10, not to be exceeded according to Fowler formula.

Inclusion criteria for patient selection were: Age ≤ 80 years; WHO performance status PS ≤ 2; PSA ≤ 20 ng/ml. Histologically proven prostate adenocarcinoma in which prophylactic lymph node irradiation is not required, (i.e. risk of microscopic involvement ≤15% or pN0 after laparoscopic pelvic node dissection in case of a risk >15%); T1-T2 stage; No pathologic lymph nodes on CT/ MRI scan; No distant metastases; No previous prostate surgery other than TURP (at least 6 weeks interval before initiation of RT); No malignant tumours in the previous 5 years; International Prostate Symptom Score (IPSS) in the range: 0–7; Combined HT according to NCCN risk factors: short term(4–6 months) neoadjuvant/concomitant/adjuvant androgen deprivation therapy (LHRH analogs and/or antiandrogens) are to propose in all intermediate risk patients. For intermediate risk, HT could be avoided in case of significant co-morbidity or when refused by the patient; Informed consent. Were considered exclusion criteria: Clinical lymph node metastasis or risk of lymph node involvement >15% [according to Roach formula for seminal vesicles]; Previous TURP less than 6 weeks before radiotherapy; Previous prostate surgery other than TURP; Previous pelvic irradiation; Inability to obtain written informed consent. Diabetes, use of anticoagulants drugs, chronic inflammatory bowel disease were considered exclusion criteria only for patients undergoing SpaceOAR™ (Augmenix, Watham, MA-USA) hydrogel injection.

### Planning and treatment details

For planning and daily treatments patients were requested to present with full bladder and empty rectum.

SpaceOAR™ hydrogel was used in selected cases as spacer to enlarge the distance between rectal wall and posterior region of prostate. SpaceOAR™ system is a synthetic hydrogel, implantable and absorbable after several months after the injection in the body; its usage was suggested in critical cases, based on physician decision based on expected potential dosimetric benefit as suggested by Weber et al. and Song et al. [3,4] and it was injected by trans-perineal injection, with trans-rectal ultrasound guide. Decision about gel implant was taken after dosimetric assessment of a treatment plan based on a CT acquired in absence of it; when plan quality was expected to be significantly improved, the gel application was proposed to the patients.

Target definition was based on CT with the support of MRI for better definition of anatomical relationships between prostate and rectum and prostate and bladder and penis bulb and to define urethra position. The clinical target volume (CTV) was considered the prostate plus entire seminal vesicles (SV) except for T1-T2 lesions with a risk of SV involvement of ≤15% in which case CTV is “prostate only”. In case of higher involvement of the SV, the first proximal third was included in the target. Planning target volume (PTV) was defined as CTV + 3-5 mm margin in each direction. During plan optimisation, the dose-volume constraints for normal tissues had priority over PTV in case of overlapping regions. Target coverage was required to be: V_95%_ > 99% on CTV (95% on PTV). Dose-volume objectives applied for dose optimisation on organs at risk (OAR) were: for rectum V_18Gy_ < 35% (50% in the first 5 patients, then constraints were tightened because technically feasible); V_28Gy_ < 10% (15%); V_32Gy_ < 5% (10%); D_1%_ < 35 Gy; for bladder D_1%_ < 35 Gy; for the other organs (femoral heads, penis bulb and healthy tissue), the strategy was to minimise the involvement as much as possible without compromising the other objectives).

The plans were designed and optimised according to the RapidArc technique with 1 or 2 full arcs with the collimator angle set to +/− 30°. Avoidance sectors were allowed to exclude direct entrance through eventual metal implants in the femoral heads. All plans were prepared to be delivered by a TrueBeam linac choosing a 10 MV flattening filter free beam. The dose rate was allowed to range up to the maximum of 2400 MU/minute.

Treatment delivery was complemented with daily image guidance by means of cone beam CT (CBCT) acquisition before each fraction delivery. If needed, couch repositioning was performed after automatic matching of CBCT images to reference planning CT, followed by manual adjustments. Fiducial markers were suggested in the protocol study for CBCT guidance to reduce set-up errors. Calcifications can be potential surrogates for prostate localization and allow for precise image guidance with a low-imaging dose [5]. Thus, we accepted to use intraprostatic calcifications as markers of prostate position: intraprostatic calcifications were identified in all 40 selected patients and exploited as reliable markers throughout the re-positioning process to drive images match. Matching was also performed on prostate gland and other soft tissue structures.

### Clinical evaluation, laboratory tests, and follow–up

Physical examination, toxicity assessment and clinical response with PSA evaluation was performed after 45–60 days following treatment or if clinically indicated. Subsequently, follow up was performed according to internal guidelines (every 3 months for the first year and every 6 months after the first year). In patients receiving hormonal treatment, we recommended testosterone levels as a surrogate for PSA. Toxicity was recorded regularly during follow-up visits, according to CTAE V.4 classification.

## Results

Descriptive data of the cohort of the 40 recruited patients of the trial were summarized in Table 1.

**Table 1:** Patient characteristics

**N. of patients**	**40**
Median Age [year]	70 [56, 80]
Median Initial PSA [ng/mL]	6.25 [0.50, 13.43]
Median Gleason Score	6 [6,7]
NCCN Low Risk Class	26
NCCN Intermediate Risk Class	14
Median F-UP [months]	10 [3-14]
N. of patients with SpaceOAR™	8

### Dosimetric data

**Figure 1:**
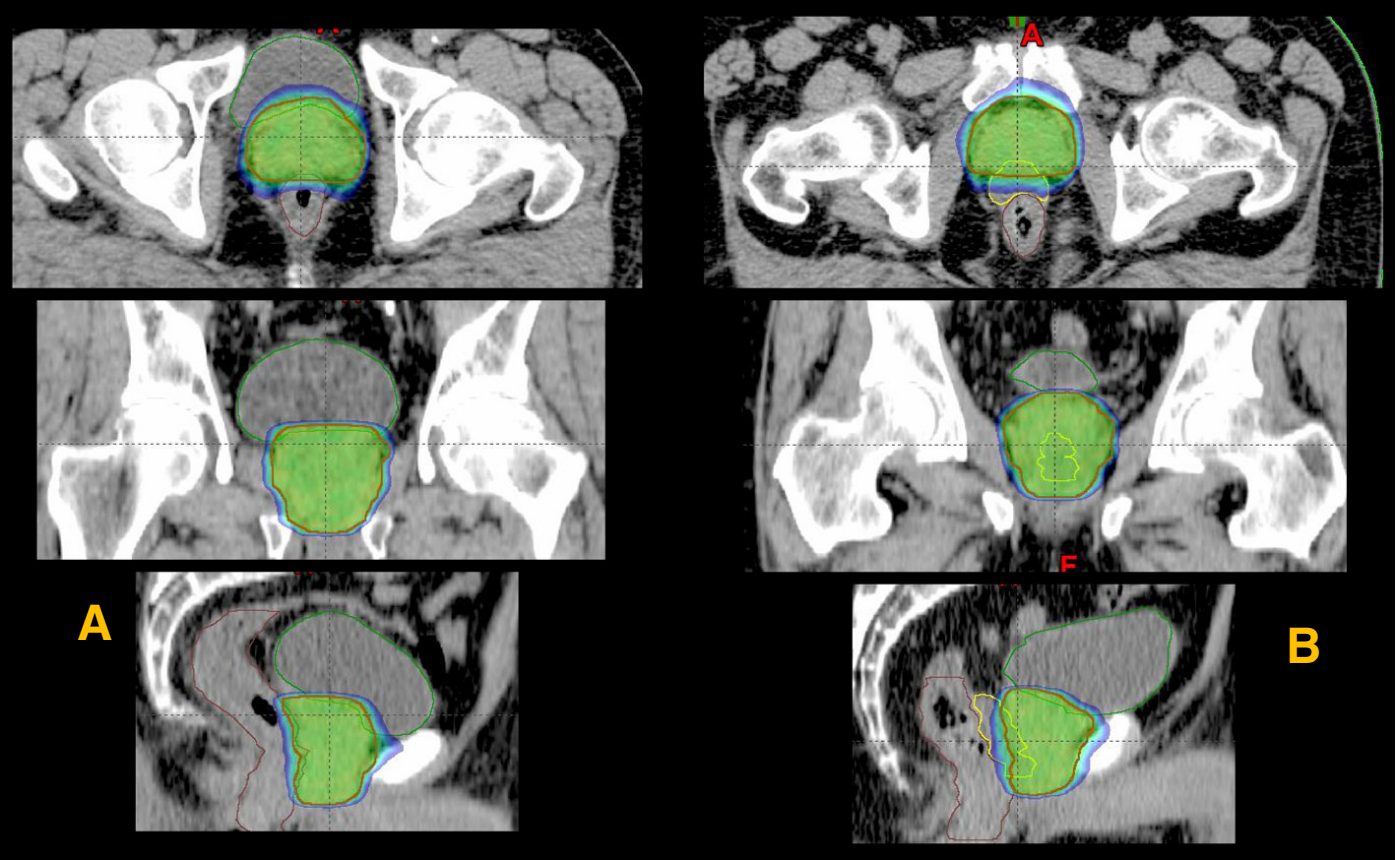
**Example of dose distributions in axial, coronal and sagittal views for one patient without (A) and one patient with SpaceOar (B).** Colorwash for dose scaling was set in the range 70-110%.

**Figure 2:**
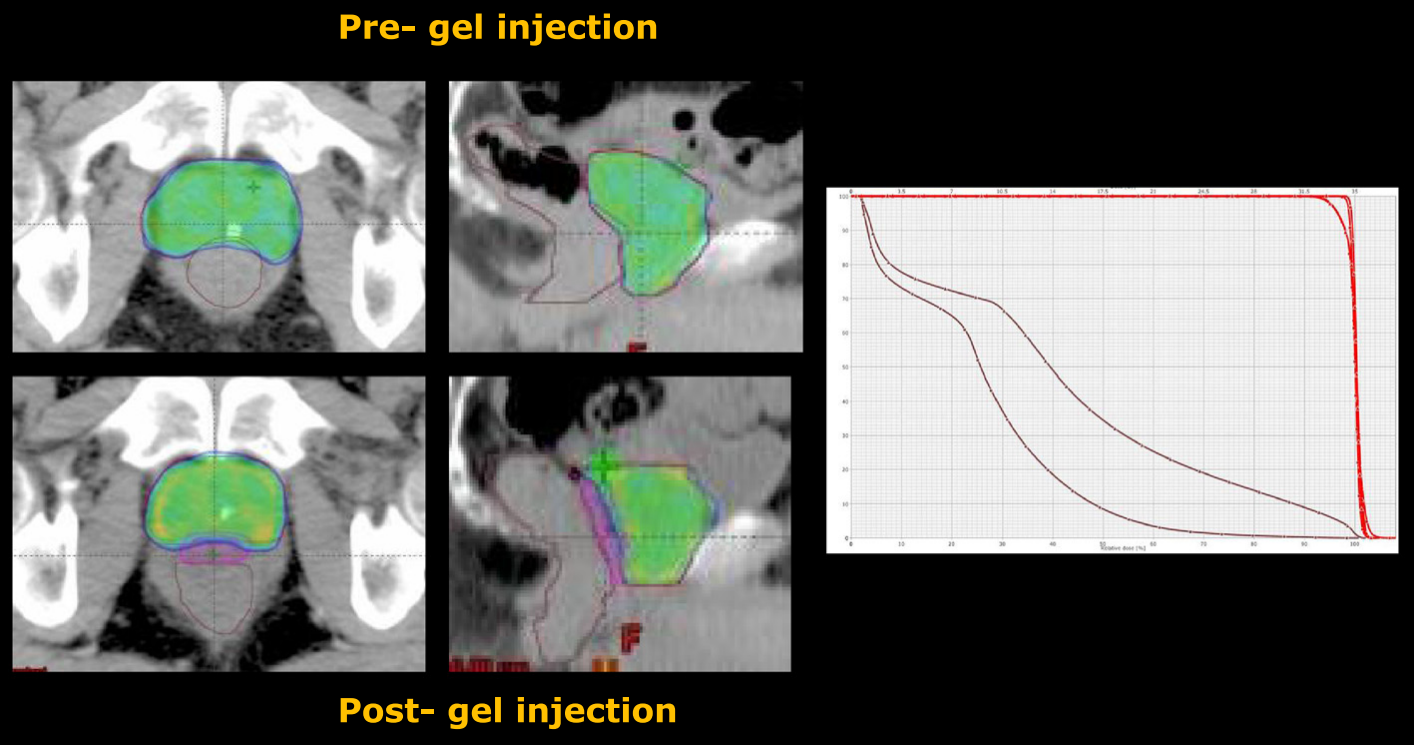
**The potential dosimetric role of SpaceOAR™ gel implantation: dose distribution and DVH for a study patient where double CT scan and double planning was performed.** Data are shown for CTV, PTV and rectum. No differences were observed for the target volumes while remarkable difference was observed in this case for the rectum.

Figure 1 presents an example of dose distributions in axial, coronal and sagittal views for one patient without (A) and one patient with SpaceOar (B). Color wash for dose scaling was set in the range 70-110%. When SpaceOAR™ gel implantation was performed, its potential dosimetric role was evaluated as shown for a study patient in Figure 2.

**Figure 3:**
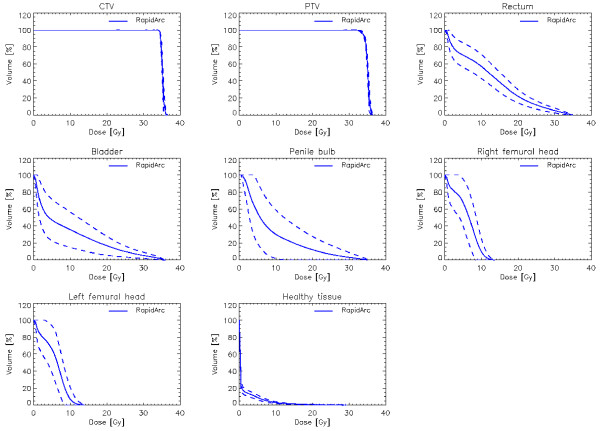
The average cumulative dose volume histograms (DVH) computed for the whole cohort of 40 patients (solid lines).

Figure 3 shows the average cumulative dose volume histograms (DVH) computed for the whole cohort of 40 patients (solid lines). The dashed lines represent the inter-patient variability expressed at + −1 standard deviation. Table 2 summarised the numerical analysis performed on CTV, PTV and OARs and based on DVHs. Reported are the main parameters valuable for plan assessment, the corresponding planning objectives, the mean values of the findings (with 1 standard deviation uncertainty) and the observed range. As it can be derived from the table, all objectives were met by all patients.

**Table 2:** Summary of the DVH analysis for the CTV, PTV and Organs at Risk

**Parameter**	**Objective**	**Mean ± SD**	**Range**
**CTV**			
Volume = 59.9 ± 21.6 cm^3^ Range = [25.1 – 110.2] cm^3^			
**Mean [Gy]**	35 Gy	35.2 ± 0.2	[34.9 - 35.8]
**V**_**95% **_**[%]**	>99%	100.0 ± 0.1	[99.4 - 100.0]
**V**_**105% **_**[%]**	Minimize	0.0 ± 0.0	-
**D**_**99% **_**[Gy]**	>33.2 (95%)	34.5 ± 0.2	[33.9 – 35.1]
**D**_**1% **_**[Gy]**	Minimize	35.9 ± 0.2	[35.5 – 36.8]
**PTV**			
Volume = 107.5 ± 33.7 cm^3^ Range = [52.8 – 182.2] cm^3^			
**Mean [Gy]**	35 Gy	35.0 ± 0.1	[34.8 – 35.7]
**V**_**95% **_**[%)**	>95%	98.3 ± 1.1	[95.6 – 99.8]
**V**_**105% **_**[%]**	Minimize	0.0 ± 0.0	-
**D**_**99% **_**[Gy]**	>31.5 (90%)	33.1 ± 0.3	[32.1 – 33.9]
**D**_**1% **_**[Gy]**	Minimize	36.0 ± 0.2	[35.6 – 36.8]
**Rectum**			
Volume = 65.7 ± 20.6 cm^3^ Range = [33.8 – 113.0] cm^3^			
**Mean [Gy]**	Minimize	12.5 ± 2.5	[6.2 – 16.1]
**D**_**1% **_**[Gy]**	<35 Gy	33.0 ± 2.4	[22.4 – 34.9]
**D**_**1cm3 **_**[Gy]**	<35 Gy	32.3 ± 2.9	[20.1 – 34.9]
**V**_**18Gy **_**[%]**	<35% (50%)	27.9 ± 9.6	[3.9 – 42.5]
**V**_**28Gy **_**[%]**	<10% (15%)	7.5 ± 3.5	[0.1 – 14.9]
**V**_**32Gy **_**[%]**	<5% (10%)	3.1 ± 1.9	[0.0 – 7.1]
**Bladder**			
Volume = 195.8 ± 118.1 cm^3^ Range = [61.5 – 544.9] cm^3^			
**Mean [Gy]**	Minimize	9.1 ± 4.4	[3.1 – 19.4]
**D**_**1% **_**[Gy]**	<35 Gy	34.1 ± 1.8	[27.5 – 35.6]
**Right femoral head**			
Volume = 73.7 ± 39.9 cm^3^ Range = [37.2 – 186.0] cm^3^			
**D**_**1% **_**[Gy]**	Minimize	10.3 ± 2.1	[4.4 – 13.8]
**Left femoral head**			
Volume = 71.8 ± 37.6 cm^3^ Range = [33.7 – 183.2] cm^3^			
**D**_**1% **_**[Gy]**	Minimize	11.3 ± 1.7	[6.4 – 14.3]
**Penile bulb**			
Volume = 4.2 ± 1.9 cm^3^ Range = [1.1 – 9.0] cm^3^			
**Mean [Gy]**	Minimize	8.9 ± 5.6	[1.2 – 21.8]
**D**_**1% **_**[Gy]**	Minimize	20.7 ± 11.2	[2.3 – 35.4]

Dx%: dose received by at least x% of the volume; Vx%: volume receiving at least x% of the dose.

Data are reported as average values plus or minus standard deviation and range.

### Clinical data: toxicity assessment

With a median follow-up of 11 months (5–16), 40 patients were recruited in the protocol and treated with the accelerated schedule of 35 Gy in 5 fractions, and were evaluable for the current analysis.

According to NCCN criteria, 26/40 patients were stratified as low-risk and 14/40 were stratified as intermediate risk. Median Age was 70 (56–80), median initial PSA was 6.25 ng/ml (0.50-13.43 ng/ml). Median Gleason score was 6 (6–7). Median treatment duration was 11.8 days (9–22). HT was prescribed in 10/40 (25%) patients, all included in NCCN intermediate risk class. All patients completed the SBRT as programmed. Median treatment time was 126 seconds (120–136).

Acute Toxicities were recorded as follow: rectum G0 in 30/40 cases (75%), G1 in 6/40 (15%); G2 in 4/40 (10%). Genito-urinary (GU) G0 in 16/40 cases (40%), G1 in 8/40 (20%), G2 in 16/34 (40%). In two G2 urinary retention cases, the placement of intermittent catheter was needed (in both cases prostate dimension was superior to 100 cm^3^). No acute G3-5 was found in the trial and 'out of trial' patients. Late toxicity (more than 6 months of follow-up) was evaluable in 25/40 trial patients. No late rectum toxicity was found; three cases of late GU G1 were found and only a case of G2 GU was experienced.

SpaceOAR™ was implanted in 8 patients with a single case of rectal fascia infection resolved with antibiotics. During Follow-up, PSA reduction was documented in all treated patients. Figure 2 shows the difference in dose distributions and DVH shape for a study patient treated with the gel and planned twice (with and without it). No differences were observed for the target volumes while remarkable difference was observed in this case for the rectum.

## Discussion and conclusions

SBRT is a promising technique that offers the ability to treat various types of solid tumours with efficacy and minimal side effects. In early non-small cell lung cancer, SBRT has yielded results comparable to those obtained with surgery, with rates of local control up to 90% in several series. Although experience to date with SBRT in prostate cancer is promising, this treatment is still in its infancy as a therapeutic option for this disease due to the small number of patients who have been treated and the relatively short follow-up [1].

In recent years, several clinical studies have employed only a few very large dose fractions, mainly in low risk, localized prostate cancer, with the aim of exploring the feasibility of such extreme hypo-fractionation schedules. A strong interest amongst the radiation oncologist community in the adoption of SBRT for localized prostate cancer has also recently prompted the Radiation Therapy Oncology Group (RTOG) to open a randomized non-inferiority Phase II trial, RTOG 0938, comparing delivery of 36.25 Gy in 5 fractions over 2 weeks to 51.6 Gy in 12 fractions over 2.5 weeks [5]. The attempt to further reduce the treatment duration in prostate cancer is based on the emulation of the HDR- brachytherapy hypo-fractionated approach in an alternative, more suitable way, allowing for steep dose gradients that resemble brachytherapy dose distributions, without the need for hospitalization, catheterization and the discomfort of keeping the delivery needles inserted for an extended time period.

Several prospective trials of extreme hypo-fractionation have already been published and several others are currently underway. The results of the more relevant SBRT trials are summarized in Table 3[7-15]. Many of these trials were carried out by the Cyberknife® and were planned to explore the feasibility of applying the shorter schedules to treat low/intermediate risk, localized prostate cancer.

**Table 3:** Summary of outcomes from SBRT trials with a follow-up of more than 30 months and at least 40 enrolled patients

**Study**	**Schedule**	**# of patients**	**Risk class**	**Medi F/U (mos)**	**Late grade 3 GU toxicity**	**Late grade 3 GI toxicity**	**FFBF**
CyberKnife							
Katz et al. 2010 [5]	35 – 36.25 Gy in 5 fx	304	L-I-H	48	2%	-	97, 93, 75% at 4 year
Freeman, King, 2011. [6]	7-7.25 Gy in 5 fx	41	L	60	< 1%	-	93% at 5 year
McBride et al. 2012 [7]	36.25-37.5 Gy in 5 fx	45	L	44.5	< 1%	-	97.7% at 3 years
Fuller et al. [8]	38 Gy in 4 fx †	54	L-I	36	4%	-	96% at 3 years
Kang et al. [9]	32-36 Gy in 4 fx	44	L-I-H	40	-	-	100%, 100%, 90.9% at 5 years
King et al. 2012 [10]	36.25 Gy in 5 fx	67	L	32.4	3.5%	-	94% at 4 years
Gantry-based Systems							
Madsen et al. 2007 [11]	33.5 Gy in 5 fx	40	L	41	-	-	90% at 4 years
Boike et al. 2011 [12]	45-50 Gy in 5 fx	45	L-I	30, 18, 12	4%	2% plus 1 Grade 4	100% at 1–2.5 years

**Abbreviations**: L = low; I = intermediate; H = high.

The premature results of these studies, although associated with good treatment tolerance, excellent early biochemical outcomes and low, late toxicity rates, did not lead to any firm conclusions on the clinical benefits of these regimens in comparison to escalated conventional dose fractionation. Noteworthy, among all hypo-fractionated regimes, very few experiences reported on acute toxicity either in the SBRT or in the moderately fractionated approach: among seven randomized trials [15-21], a detailed description of acute toxicity was reported only in two trials [21,22]. Differently from late toxicity, acute rectal toxicity occurs during or within 3 months after completion of treatment and is temporary. However, the acute effects may be severe enough to interrupt the planned course of treatment in 10% of the patients. In addition, with conventional fractionation regimes, a high rate of acute rectal toxicity is now recognized to be associated with late proctopathy [23]. Both studies reported a slightly higher but not statistically significant rate of grade 2 or more GI and GU acute toxicity in the short over the long treatment arms, with an earlier peak for both rectal and urinary toxicity in the former arm. Arcangeli et al. [22], found that the median interval to toxicity detection was 22 and 36 days (p = 0.001) for GI and 15 and 23 days (p = 0.002) for GU in the hypo-fractionation and conventional fractionation arm, respectively, but no difference in the duration of either GI or GU toxicities was encountered between the two treatment schedules (p = 0.31 and 0.34, respectively).

In the present study, a result to underline is the absence of G3 or greater toxicity in GU and rectum. The relevance of this finding is significant, if related to the fact that this is the first experience where FFF delivered SBRT has been tested for prostate cancer patients.

The main limit of the present report remains the short follow-up (approximately 11 months), un-sufficient to determine late toxicity and FFBF rates. Nevertheless, toxicity reported with 8 months or greater of follow-up, evaluable in 25 of 40 patients, seems to confirm the optimal preliminary tolerability of the treatment approach: only three cases of late GU G1, a case of G2, and no cases of late rectum toxicity has been reported. Given the absence of relapses in the treated patients, also preliminary data on biochemical control seems to be promising and in line with those published in other series, as shown in Table 3.

The current 3 to 4-year FFBF rates of >90%, reported in all SBRT published trials with a sufficient follow-up [7-15], seems to be consistent with the 5-year rates of ~90%–95% reported in trials of conventional escalated doses of 78-80 Gy. However, given the uncertainties, which exist in extrapolating biological effects to very large fraction size, these results need to be confirmed by appropriate randomized trials with a sufficiently long follow-up and accurate evaluation of long term tolerance and toxicity, particularly of the urethra which is an unavoidable organ at risk in the irradiation of prostate cancer.

Another possible criticism of the present study regards the use of SpaceOAR™. The hydrogel spacer was implanted, based on physician decision, only in a limited subgroup of trial patients, considered critical. We are conscious on the subjectivity of this choice. However, in the absence of general consensus, the institutional experience suggests that the use of the spacer might be beneficial from the dosimetric viewpoint, in a subgroup of patients to be accurately selected.

In summary, our early findings suggest that LINAC based SBRT FFF treatment for prostate cancer in 5 fractions is feasible, fast and well tolerated in acute setting. Longer follow-up is needed for definitive assessment of late toxicity and clinical outcome.

## Competing interests

Dr. Filippo Alongi: Varian Honoraria, Augmenix Honoraria.

Dr. L. Cozzi acts as Scientific Advisor to Varian Medical Systems and is Head of Research and Technological Development to Oncology Institute of Southern Switzerland, IOSI, Bellinzona. The study was partially financed by a research grant of Varian Medical systems. Other Conflict of Interest: None.

## Authors’ contributions

MS, FA, TC, AT, LC designed the study and the analysis. PN, CI, EC, LR, AZ, MS, AT, TC, FA collected the clinical data, PM, GR, ST collected the dosimetric data. AT, TC, ST, LC, AFC performed main data analysis. AT, TC, FA, LC drafted the manuscript. All authors reviewed and approved the final manuscript.
